# Functional endemism: population connectivity, shifting baselines, and the scale of human experience

**DOI:** 10.1002/ece3.446

**Published:** 2012-12-19

**Authors:** Joshua Drew, Les Kaufman

**Affiliations:** 1 Department of Ecology, Evolution and Environmental Biology, Columbia University1200 Amsterdam Ave., New York, NY, 10027; 2 Biology Department, Boston University5 Cummington Street, Boston, MA, 02215; 3 Conservation International2011 Crystal Drive, Arlington, Virginia, 22202

**Keywords:** Coral Reefs, historical ecology, migrants per generation, population connectivity, shifting baselines

## Abstract

Quantifying population connectivity is important for visualizing the spatial and temporal scales that conservation measures act upon. Traditionally, migration based on genetic data has been reported in migrants per generation. However, the temporal scales over which this migration may occur do not necessarily accommodate the scales over which human perturbations occur, leaving the potential for a disconnect between population genetic data and conservation action based on those data. Here, we present a new metric called the “Rule of Memory”, which helps conservation practitioners to interpret “migrants per generation” in the context both of human modified ecosystems and the cultural memory of those doing the modification. Our rule states that clades should be considered functionally endemic regardless of their actual taxonomic designation if the migration between locations is insufficient to maintain a viable population over the timescales of one human generation (20 years). Since larger animals are more likely to be remembered, we quantify the relationship between migrants per human (N) and body mass of the organism in question (M) with the formula N = 10M^−1^. We then use the coral reef fish *Pomacentrus moluccensis* to demonstrate the taxonomic and spatial scales over which this rule can be applied. Going beyond minimum viable population literature, this metric assesses the probability that a clade's existence will be forgotten by people throughout its range during a period of extirpation. Because conservation plans are predicated on having well-established baselines, a loss of a species over the range of one human generation evokes the likelihood of that species no longer being recognized as a member of an ecosystem, and thus being excluded in restoration or conservation prioritization. [Correction added on 26 December 2012, after first online publication: this formula has been corrected to N=10M^−1^].

## Introduction

Understanding population connectivity is an important component in establishing effective conservation strategies (reviewed in Rocha et al. 2007). By delineating the relationship between two populations as expressed in the number of migrants exchanged per time interval, and by identifying asymmetries within that migration, we are able to describe the degree to which these populations are mutually supportive, reinforcing and evolving together. Conversely, identifying barriers to population connectivity can help managers to appropriately distribute limited resources in order to maximize population resilience and reduce a species' vulnerability to extirpation (Botsford et al. 2009).

Population connectivity has been expressed through a variety of measures including F statistics (Drew et al. 2008; Eble et al. 2010), and their corollary, effective numbers of migrants, (Bay et al. 2008; Drew and Barber 2009) and assignment tests (Burford and Bernardi 2008; Saenz-Agudelo et al. 2009). Genetic assessments of connectivity are useful because they provide time-averaged measures and are less susceptible to influences from episodic recruitment (Wiegand et al. 2004; Chabanet et al. 2005) Such episodic phenomena can produce errant extrapolations from other methods, such as biogeochemistry (Thorrold et al. 2001), that focus on snapshots of cohorts and not on long-term population dynamics.

Population genetic theory (Slatkin 1987) and simulations (Mills and Allendorf 1996; Wang 2004) show that as little as one migrant per generation is necessary to prevent fixation. We note that this estimate is founded in a variety of simplifying assumptions and, in some cases, substantially higher migration rates may be required to prevent fixation (Vucetich and Waite 2000). Regardless, the one migrant per generation rule of thumb has proven an extremely useful benchmark in studies focusing on the evolutionary distinctness of populations (Gonzales et al. 1998; Newman and Tallmon 2001). However, evolutionary cohesiveness and ecological connectivity function at two very different temporal scales and migration which is sufficient to provide evolutionary stability may not provide enough individuals to insure ecological rescue from perturbations.

This disconnect between the time scales of population connectivity and the time scales of human influences hinders conservation management because (1) Human perturbations to the environment often occur on the scales of a few years to decades; time periods shorter than the generation times of long lived species; (2) Human impressions of what a natural ecosystems consists of are highly dependent on the conditions that people initially experience, and rarely are these conditions relayed over more than four human generations (Pauly 1995; Baum and Myers 2004; Knowlton and Jackson 2008; McClenachan 2009a). How population connectivity (as a function of ecosystem resilience or restoration) influences the shifting baseline syndrome needs to be evaluated over periods of 20 years – one human generation.

The shifting baseline syndrome (Pauly 1995) chronicles the change in perception of natural systems toward an increasingly diminished state. This cultural amnesia is brought about by each generation accepting the environment as “natural” regardless of the condition that it is in. Over time, this leads to people accepting less diverse ecosystems (Knowlton and Jackson 2008) with reductions in predator size (McClenachan 2009b) or community diversity (Baum and Myers 2004). This phenomenon often manifests itself in intergenerational differences in perception of the ecosystem quality (Ainsworth et al. 2008; Bunce et al. 2008), community composition (Turvey et al. 2010), or lower trophic level of harvested species (Alexander et al. 2011). The key similarity in these studies is that changes to the ecosystems occur on the time span of one human generation. Shifts occurring within a generation will be manifested as differences in how generations set their expectations of what an ecosystem should look like. Failure to transmit any sort of transgenerational information (Drew 2005) will result in an inexorable shift toward accepting an altered ecosystem as “normal”

## Estimating migrants per generation in a human context

We propose that in order to measure the minimum amount of migration necessary to forestall the shifting baseline syndrome, it is necessary to calculate the number of migrants per human generation (20 years). This calculus requires both accurately estimating the number of migrants per generation as well as quantifying the generational time of the species in question. Neither of these is a trivial task.

While it is possible to calculate migrants per generation directly from F_st_ values, Palumbi (2003) correctly points out that the relationship between migrants per generation and F_st_ is asymptotic, so that determining an accurate estimation of migrants at low levels of F_st_ is problematic. For example, at extremely low values, the measurement error in F_st_ can produce estimates of N_e_m that vary by several orders of magnitude (Palumbi 2003). Furthermore, spatial heterogeneity can violate the underlying assumption of Wright's model – that each subpopulation has an equal chance of contributing to the migrant pool (Wright 1931). Violations of the assumption of no spatial structure can lead to an increase in F_st_ estimations when there is high turnover among populations as founding individuals often contain only a small subset of the total variation within a species (reviewed in Whitlock and McCauley 1999). A third potentially confounding factor is the difference between actual migrants and effective number of migrants. All migrants are not created equal. Should migrants fail to reproduce, they will factor in the F_st_/N_e_m calculus. In some cases, new migrants face significant challenges to joining the gene pool. These can include lack of locally adapted gene combinations (Via 2009), incongruence in mating recognition leading to prezygotic isolation, or preferential predation on novel behaviors or coloration (Langham 2007)

Generation time can be calculated using the formula *t* = (*α* + *ω*)/2 (van Herwerden and Doherty 2006), where *α* is age at first reproduction and *ω* is longevity. These numbers can be influenced by a suite of species-specific factors that underscore the importance of understanding the natural history of the organism in question. For example, species which have socially stratified access to mates vary at, the age at which males and females breed. Similarly, flexible mating strategies in species (such as altering between haremitic and pair-based systems of mating based on resource availability) may result in ecologically mediated differences in generation time. Finally, longevity can be very difficult to calculate and estimates from congeners or from captive-reared specimens may have to suffice in the case where more specific information is unavailable (Miller et al. 2002).

Despite these technical difficulties, increases in sequencing technology and advances in both the sophistication and computational power of migration estimation algorithms are making the accurate estimation of the numbers of migrants possible even for a modestly equipped laboratory. In general, an increase in the number of unlinked loci used helps to reduce the distribution of the migration prior in a Bayesian framework (Beerli 2006; Heled and Drummond 2008), or to help improve likelihood measurements (Felsenstein 2006) in a Maximum Likelihood environment because they often effectively serve as independent trials of population genetic hypotheses. With local advances in developing unlinked nuclear loci, it is now possible to develop robust data sets to help estimate the time, number, and direction of migrations (Leaché 2011), especially when these are considered using non-equilibrium models of isolation and migration (Balakrishnan and Edwards 2009; Marko and Hart 2011).

## Conservation implications: The rule of memory

With the advent of molecular taxonomy, we have found that many species previously thought to have broad geographic ranges actually are composed of several geographically restricted distinct evolutionary groups. Termed “cryptic species,” this evolutionary phenomenon is widespread across both the globe and the metazoan lineage (Pfenninger and Schwenk 2007). The impacts of cryptic diversity on conservation (Knowlton 1993; Rubinoff 2006; Bickford et al. 2007) and on taxonomy (Fukami et al. 2004; Will and Rubinoff 2004; Mace and Holdon 2005; Drew et al. 2008) are well known, but here we specifically link the ideas of population connectivity, conservation biology, and endemism using coral reef fishes (our area of expertise) as a case study.

Until recently, coral reef fishes of apparently broad distribution have been considered conspecific throughout the Indo-Pacific, from East Africa to the central or eastern Pacific Ocean – often despite considerable regional variation in coloration, and sometimes in meristics as well. In a few instances, genetic studies suggest sufficient dispersal to render a taxon panmictic throughout a broad or even global range (Craig et al. 2007; Theisen et al. 2008). In other cases, circumtropical ring species have been demonstrated, as in the *Aulostomus maculatus/chinensis/strigosus* (Bowen et al. 2001). We suggest, however, that in tropical shore fishes allopatric semispecies (sensu Mayr 1940) characterized by distinct, evolutionarily isolated clades of common ancestry, with each form endemic to a continental margin or island group, may be more the rule than the exception. We have already seen a similar phenomenon within several independent lineages of marine invertebrates (Meyer et al. 2005; Malay and Paulay 2010), and it may prove to be more common in fishes as we continue to sample more intensively (Drew et al. 2008, 2010). This pattern of archipelagic endemism has implications for marine conservation strategy, which must clearly be geared toward evolutionary stable units rather than superficially coherent superspecies. But where should the line be drawn? The question has two dimensions: evolutionary and socio-cultural. In an evolutionary sense, a search for a moment of speciation is perhaps subjective. In general, species can be thought of as populations that are coupled on the same evolutionary trajectory (Hey and Pinho 2012). Despite the sharp discontinuity that eventually forms between daughter species, during the process of speciation itself, metapopulation structure and selection together weave an information-rich and seemingly continuous spatial and temporal tapestry. The result is that definitive rules for just when daughter clades constitute separate species are difficult to craft.

In sociocultural terms, the conditions for recognizing species as distinct are much clearer, although rarely articulated (Begossi et al. 2008). When viewing taxonomy from a conservation perspective, the issue becomes how a clade is likely to fare in the current anthropogenic mass extinction event. In other words, can it survive in the face of anthropogenic influences on the biosphere and direct take from wild populations? Unless a clade merits species recognition and is given a name, options for its conservation are very limited for reasons that are psychological, cultural, and legal. This is not of itself a valid justification for bumping regional populations up to all be named as species. We propose that a key criterion should be the probability that a clade's very existence will be forgotten by people throughout its range, and a life form thus inadvertently destroyed due to ignorance or negligence.

This probability is subject to the amount of quality data available. In an ideal case, we will have long-term documentation of ecosystem changes (Alexander et al. 2009); however, in many cases, the written record will be substantially less dense, and alternative forms of data, including natural history museum collections, must be used (Drew 2011; Hoeksema et al. 2011). In many cases, however, there will be no written record and for highly localized investigations, we must rely on the collective cultural memory of the people living in a particular area.

In terms of population biology, this probability means that there must be a chance for population rescue within the memories, or at very least the lifetimes, of individuals. An average human generation is 20 years, thus in order for populations to exist within the generational memory, they must exchange enough migrants to provide a visible presence every (human) generation. This benchmark is not the minimum viable population of either the population in question, or the metapopulation as a whole, which can often be substantially higher (Traill et al. 2008). Rather, our benchmark is the minimum number of individuals necessary for local (human) populations to recognize that a particular species is part of the natural community. We furthermore suggest that the number of individuals necessary should be inversely related to the biomass of the organism – a whale shark is going to be far more memorable than a damselfish after all. We suggest therefore that as a guideline to frame this discussion, the number of migrants to forestall the shifting baseline syndrome be:


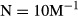
(1)

where M is the average adult weight in kilograms of the organism in question, and N is the number of individuals that need to be observed per human generation (20 years) in order to retain that species in the cultural memory of individuals living in a unit of area. Furthermore, we suggest there should be a minimum of one migrant per three human generations (60 years) as this a typical generational span found in human communities. The latter point is especially important for large bodied organisms, which are likely to remain in the collective consciousness (Turvey et al. 2010).

The rule of memory is spatially dependent and is reliant, in part, on the spatial scales over which the genetic data were captured. Studies with more fine scale genetic sampling are more likely to determine subtle geographic shifts in population structure that in turn may lead to more spatially nuanced applications to the rule of memory.

## Worked Example: Gene flow among populations of *Pomacenturs moluccensis*

To see this benchmark applied to a real-world example, we use data from a common Indo-Pacific coral reef fish *Pomacentrus moluccensis*. This species weighs approximate 5 g (Nilsson and Östlund-Nilsson 1997) and reproduces within 1 year and has a longevity of 8 years (Waldie et al. 2011) for a generation time of 4.5 years. On the basis of our formula, we would need 2000 migrants per human generation, or approximately 100 per year to maintain this fish within a groups' collective memory. These fish are often found in schools of 10–30 individuals on branching coral heads, thus the level of connectivity needed for our threshold would involve the introduction of approximately 3–10 colonies of fish per year, hardly a major influx of individuals, and almost certainly below what the minimum viable population would require.

Drew and Barber (2009) calculated migration rates for several populations of *P. moluccensis* across the Indo-Pacific and the Southwest Pacific. Using their results and, given our benchmark of 100 fish per year, the populations of Fiji (20.1 median average number of migrants per fish generation or ∼4.5 fish per year exchanged with nearest neighbor Vanuatu) and Vanuatu (9.8 individuals per fish generation or ∼2.2 fish/year) are both functioning as endemics, a result further borne out by their phylogeny. Recently, Allen and Drew (2012) elevated the Fijian population of *P. moluccensis* to a full species status, *P. maafu* in part due to the genetic divergence among the populations.

When considering the more taxonomically ambiguous situation looking between the migration between the phenotypically identical Vanuatu population and their neighbors to the west in Papua New Guinea and Indonesia, one finds that with one expectation no proximal populations are exchanging sufficient migrants to meet our threshold (Drew and Barber 2009). The only exception being the exchange from the Eastern Indonesian Islands and Papua New Guinea to the Central Indonesian Islands (*N* = 171 fish per year)

In contrast, Drew and Barber (2012) examined gene flow among populations of *P. maafu* (the recently described Southwest Pacific endemic species previously recognized as *P. moluccensis*) within and among four regions of Fiji. In this study, they found that in three of twelve possible pairwise comparisons, there was sufficient exchange of larvae as to forestall the shifting baseline syndrome

## Discussion and Conclusions

We propose that a regional population is performing as an extant species in all respects including in its interaction with human society, if its extirpation is unlikely to be reversed by population rescue from afar within 20 years. This is the minimum level of connectivity that would, for instance, allow a damaged reef to be restored to post-perturbation conditions within a time span dictated by the generation of people living in association with that reef. Species with migration rates less than this level run the risk of being erased from a culture's memory. Associated with this cultural amnesia would be a consistent biological erosion toward a duller and more degraded state (Pauly 1995; DeMartini et al. 2008).

We call this the “Rule of Memory”. In other words, if the odds are that a taxon will be declared locally extinct, or its presence entirely forgotten following extirpation due to a low likelihood of population rescue, then it should be recognized as a distinct species endemic to that locale. This idea is consistent with the concept of an “Evolutionarily Stable Unit” as applied under the US Endangered Species Act, but goes beyond it in frankly considering aspects of human behavior with regard to an endangered taxon.

The rule of memory is sensitive to the quality of the data used to evaluate it. First, it requires accurate estimations of generation lengths. When these are not available, researchers are forced to extrapolate from congeners. Second exploitation has several well-documented impacts on individual life histories, including smaller age of first reproduction (Conover et al. 2009), smaller size, and subsequent home range size (Shackell et al. 2012a,b), thus there may be substantive changes in the biological characteristics of the organism in question occurring within the three generation window. Lastly, one needs to consider the knowledge of the observer. Several studies have demonstrated the use of knowledgeable fisher interviews (Sáenz–Arroyo et al. 2005; Ainsworth et al. 2008; Boudreau and Worm 2010). When relying on only oral reports, we suggest placing greater emphasis on individuals who have a long-term intimate knowledge of the area (Drew 2005).

Furthermore, while we focus on biomass as being the main driver in our calculations, we can easily envision the formula being expanded to include differences in coloration (by definition, cryptic species are less likely to be observed), depth (deeper species are less likely to be observed), or behavior (diurnal species being far more visible). We encourage other researchers to expand our research and help refine the calculations underlying our Rule of Memory. This desire for an ongoing dialogue with researchers, managers, and conservation practitioners from around the world was one of the major drivers for us to publish in an open access format.

Some will find the Rule of Memory, and indeed the entire notion of a “conservation” threshold for species recognition, to be capricious. We argue that it is an essential instrument in the battle against mass extinction. Coupled with strong endangered species laws, institution of the Rule of Memory can reduce the loss of cryptic marine species and arrest the erosion of marine biodiversity around the world. We further suggest that the Rule of Memory be considered for all taxa, marine and terrestrial.
